# Supplementary material for: HIVprotI: an integrated web based platform for prediction and design of HIV proteins inhibitors
Source: J Cheminform. 2018 Mar 9;10:12. doi: 10.1186/s13321-018-0266-y (PMC5845081; doi:10.1186/s13321-018-0266-y)
Supplement: Supplementary file 2 — Additional file 2. Source code of HIVProtI web server. [file 13321_2018_266_MOESM2_ESM.zip › HIVprotI_Source-code/hivprotI/weka-3-6-6/doc/weka/classifiers/trees/j48/NoSplit.html]

NoSplit


---


|  |  |  |
| --- | --- | --- |
| **Overview** \| **Package** \| **Class** \| **Tree** \| **Deprecated** \| **Index** \| **Help** \| **Weka's home** | |  |
| **PREV CLASS**   **NEXT CLASS** | **FRAMES**    **NO FRAMES**     **All Classes** |  |
| SUMMARY: NESTED \| FIELD \| CONSTR \| METHOD | DETAIL: FIELD \| CONSTR \| METHOD |  |


---


## weka.classifiers.trees.j48 Class NoSplit

```
java.lang.Object
  weka.classifiers.trees.j48.ClassifierSplitModel
      weka.classifiers.trees.j48.NoSplit
```

**All Implemented Interfaces:**: java.io.Serializable, java.lang.Cloneable, RevisionHandler

---

``` public final class NoSplit extends ClassifierSplitModel ```

Class implementing a "no-split"-split.

**Version:**
:   $Revision: 1.9 $

**Author:**
:   Eibe Frank (eibe@cs.waikato.ac.nz)

**See Also:**: Serialized Form

---

**Constructor Summary**

`NoSplit(Distribution distribution)`
  
          Creates "no-split"-split for given distribution.


| **Method Summary** | |
| --- | --- |
| `void` | `buildClassifier(Instances instances)`             Creates a "no-split"-split for a given set of instances. |
| `java.lang.String` | `getRevision()`             Returns the revision string. |
| `java.lang.String` | `leftSide(Instances instances)`             Does nothing because no condition has to be satisfied. |
| `java.lang.String` | `rightSide(int index, Instances instances)`             Does nothing because no condition has to be satisfied. |
| `java.lang.String` | `sourceExpression(int index, Instances data)`             Returns a string containing java source code equivalent to the test made at this node. |
| `double[]` | `weights(Instance instance)`             Always returns null because there is only one subset. |
| `int` | `whichSubset(Instance instance)`             Always returns 0 because only there is only one subset. |

**Methods inherited from class weka.classifiers.trees.j48.ClassifierSplitModel**

`checkModel, classifyInstance, classProb, classProbLaplace, clone, codingCost, distribution, dumpLabel, dumpModel, numSubsets, resetDistribution, sourceClass, split`

**Methods inherited from class java.lang.Object**

`equals, getClass, hashCode, notify, notifyAll, toString, wait, wait, wait`

**Constructor Detail**

### NoSplit

```
public NoSplit(Distribution distribution)
```

:   Creates "no-split"-split for given distribution.


**Method Detail**

### buildClassifier

```
public final void buildClassifier(Instances instances)
                           throws java.lang.Exception
```

:   Creates a "no-split"-split for a given set of instances.

    :   **Specified by:**: `buildClassifier` in class `ClassifierSplitModel`
    :   **Throws:**: `java.lang.Exception` - if split can't be built successfully

---


### whichSubset

```
public final int whichSubset(Instance instance)
```

:   Always returns 0 because only there is only one subset.

    :   **Specified by:**: `whichSubset` in class `ClassifierSplitModel`

---


### weights

```
public final double[] weights(Instance instance)
```

:   Always returns null because there is only one subset.

    :   **Specified by:**: `weights` in class `ClassifierSplitModel`

---


### leftSide

```
public final java.lang.String leftSide(Instances instances)
```

:   Does nothing because no condition has to be satisfied.

    :   **Specified by:**: `leftSide` in class `ClassifierSplitModel`
    :   **Parameters:**: `instances` - the data.

---


### rightSide

```
public final java.lang.String rightSide(int index,
                                        Instances instances)
```

:   Does nothing because no condition has to be satisfied.

    :   **Specified by:**: `rightSide` in class `ClassifierSplitModel`

---


### sourceExpression

```
public final java.lang.String sourceExpression(int index,
                                               Instances data)
```

:   Returns a string containing java source code equivalent to the test
    made at this node. The instance being tested is called "i".

    :   **Specified by:**: `sourceExpression` in class `ClassifierSplitModel`
    :   **Parameters:**: `index` - index of the nominal value tested: `data` - the data containing instance structure info **Returns:**: a value of type 'String'

---


### getRevision

```
public java.lang.String getRevision()
```

:   Returns the revision string.

    :   **Returns:**: the revision


---


|  |  |  |
| --- | --- | --- |
| **Overview** \| **Package** \| **Class** \| **Tree** \| **Deprecated** \| **Index** \| **Help** \| **Weka's home** | |  |
| **PREV CLASS**   **NEXT CLASS** | **FRAMES**    **NO FRAMES**     **All Classes** |  |
| SUMMARY: NESTED \| FIELD \| CONSTR \| METHOD | DETAIL: FIELD \| CONSTR \| METHOD |  |


---
